# Inconsistencies of the Evaluation of Home Advantage in Sports Competitions Under the Three Points Per Victory System

**DOI:** 10.2478/hukin-2014-0055

**Published:** 2014-10-10

**Authors:** Miguel Saavedra García, Óscar Gutiérrez Aguilar, Juan J. Fernández Romero

**Affiliations:** 1 University of A Coruña (Spain).; 2 University Miguel Hernández (Spain).

## Abstract

A recent letter sent to the Journal of Human Kinetics’ editor (Gómez & Pollard, 2014) warned about a supposed methodology error in the calculation of home advantage in football leagues used in Saavedra et al. (2013) and took the liberty of modifying the research’s data. The aim of this letter is to demonstrate that the evaluation system of the home advantage proposed by Pollard (1986) contains serious inconsistencies when applied to competitions which give three points for a win and one point for a draw, as it is the case of the UEFA football leagues in the 21th century.

The analysis of home advantage (HA) aims to determine the existence of a benefit to the local teams and must meet the following axioms:

- Two teams which obtain the same amount of points as local must have the same HA value.
- Points obtained as local and HA must have a direct relationship, this is, the more/less amount of points obtained as local team, the more/less value of HA (as long as the number of matches played is constant).

These axioms meet in the Pollard’s model (1986) when this is applied to competitions which give two points for a win and one point for a draw, but they do not meet in competitions which give three points for a win and one for a draw.

For those competitions which do not allow a draw, Pollard (1986) suggests that the value of HA must be calculated as the number of matches won as local team and expressed as a percentage out of the total number of matches played. If the competition gives three points for a win and one for a draw, Pollard (1986) seems to ignore the effect caused, since a point per match ended in a draw is casted aside to calculate the total points obtained, which affects the HA value. This is avoided in Saavedra et al. (2013).

When Pollard (1986) applies his theoretical model, he does not use a stable criterion, since there is a lack of justification for having excluded the matches which end in a draw in the US football and cricket and not in the rest of sports which allow ending in a draw too.

The *first inconsistency* lies in the fact that two different competitions with the same number of matches and the same number of points obtained at home can have a different HA value when the Pollard’s model (1986) is applied. This fact is illustrated (Table 1) by using the data of the study by Sánchez et al. (2009).

The *second inconsistency* in the Pollard’s model (1986) lies in the fact that HA does not have a direct relationship with the number of points obtained as a local team (as long as the same number of matches is played). This problem is solved by following the model suggested by Saavedra et al. (2013). This fact is illustrated (Table 2) by using the data of the study by Sánchez et al. (2009).

## Figures and Tables

**Table 1:** Two teams which obtain the same number of points as a local team will obtain the same HA value.

Second division	Number of games	Home wins	Away wins	Draws	Home points	HA Pollard (1986)	HA Saavedra et al (2013)^*^
1998–99	462	206	124	132	750	59,81	54,11
1999–00	462	203	118	141	750	60,24	54,11

*Data* from Sánchez et al. (2009), with the exception of the entries marked with^*^.

**Table 2:** Points obtained as a local team and HA must have a direct relationship.

Season	Division	Number of games	Points per win	Points gained home teams	HA Sánchez et al. (2009)	HA Saavedra et al. (2013)^*^
1997–98	1	380	3	656	63,69	57,54
1999–00	1	380	3	658	64,01	57,72
2001–02	1	380	3	659	63,43	57,81
1998–99	1	380	3	670	64,24	58,77
2004–05	1	380	3	676	65,00	59,30
2000–01	1	380	3	696	66,86	61,05
1996–97	1	380	3	779	61,48	68,33

Data from Sánchez et al. (2009), with the exception of the entries marked with^*^.
